# Use of a Wearable Biosensor to Study Heart Rate Variability in Chronic Obstructive Pulmonary Disease and Its Relationship to Disease Severity

**DOI:** 10.3390/s22062264

**Published:** 2022-03-15

**Authors:** Seon-Cheol Park, Narongkorn Saiphoklang, Donghyun Jung, David Gomez, Jonathan E. Phillips, Brett A. Dolezal, Donald P. Tashkin, Igor Barjaktarevic, Christopher B. Cooper

**Affiliations:** 1 Division of Pulmonary, Critical Care and Sleep Medicine, Department of Medicine, David Geffen School of Medicine, University of California Los Angeles, Los Angeles, CA 90095, USA; parksc@nhimc.or.kr (S.-C.P.); m_narongkorn@hotmail.com (N.S.); bdolezal@mednet.ucla.edu (B.A.D.); dtashkin@mednet.ucla.edu (D.P.T.); ibarjaktarevic@mednet.ucla.edu (I.B.); 2 Division of Pulmonology, Department of Internal Medicine, National Health Insurance Service Ilsan Hospital, Goyang 10444, Korea; 3 Department of Medicine, Faculty of Medicine, Thammasat University, Pathum Thani 10120, Thailand; 4 UCLA Airways and Exercise Physiology Research Laboratory, David Geffen School of Medicine, University of California Los Angeles, Los Angeles, CA 90095, USA; djung@mednet.ucla.edu (D.J.); davidgomez144@gmail.com (D.G.); 5 Inflammation Discovery Research, Amgen, Thousand Oaks, CA 91320, USA; jphill01@amgen.com; 6 Department of Physiology, David Geffen School of Medicine, University of California Los Angeles, Los Angeles, CA 90095, USA

**Keywords:** heart rate variability, wearable sensors, chronic obstructive pulmonary disease, bronchodilator, health

## Abstract

The purpose of this study was to explore the relationships between heart rate variability (HRV) and various phenotypic measures that relate to health and functional status in chronic obstructive pulmonary disease (COPD), and secondly, to demonstrate the feasibility of ascertaining HRV via a chest-worn wearable biosensor in COPD patients. HRV analysis was performed using SDNN (standard deviation of the mean of all normal R-R intervals), low frequency (LF), high frequency (HF), and LF/HF ratio. We evaluated the associations between HRV and COPD severity, class of bronchodilator therapy prescribed, and patient reported outcomes. Seventy-nine participants with COPD were enrolled. There were no differences in SDNN, HF, and LF/HF ratio according to COPD severity. The SDNN in participants treated with concurrent beta-agonists and muscarinic antagonists was lower than that in other participants after adjusting heart rate (beta coefficient −3.980, *p* = 0.019). The SDNN was positively correlated with Veterans Specific Activity Questionnaire (VSAQ) score (*r* = 0.308, *p* = 0.006) and handgrip strength (*r* = 0.285, *p* = 0.011), and negatively correlated with dyspnea by modified Medical Research Council (mMRC) questionnaire (*r* = −0.234, *p* = 0.039), health status by Saint George’s Respiratory Questionnaire (SGRQ) (*r* = −0.298, *p* = 0.008), symptoms by COPD Assessment Test (CAT) (*r* = −0.280, *p* = 0.012), and BODE index (*r* = −0.269, *p* = 0.020). When measured by a chest-worn wearable device, reduced HRV was observed in COPD participants receiving inhaled beta-sympathomimetic agonist and muscarinic antagonists. HRV was also correlated with various health status and performance measures.

## 1. Introduction

Heart rate variability (HRV) is a means of evaluation of the influence of the autonomic nervous system (ANS) on control of heart rate. It reflects the balance of sympathetic and parasympathetic influences, with any increase in sympathetic stimulation and/or decrease in parasympathetic (vagal) stimulation reduced HRV. There are published standards regarding the measurement and interpretations of HRV [1].

HRV has been reported to offer prognostic information in a variety of diseases, such as respiratory failure, diabetes, renal failure, cirrhosis, cancer, and depression [2,3,4,5,6]. Reduced HRV has been associated with an increase in cardiovascular events and mortality in population-based studies [7,8].

Chronic obstructive pulmonary disease (COPD) is characterized by respiratory symptoms together with air flow limitation and is a major cause of chronic morbidity and mortality [9,10]. HRV has been reported to be reduced in patients with COPD [11,12,13,14]. This could be due to various mechanisms: disease progression, general decline in health status, deconditioning or medications used to treat COPD, and specifically inhaled bronchodilators.

The main therapy for stable COPD is inhaled bronchodilators such as β-agonist or muscarinic antagonist [15,16,17]. These medications may be potential contributors to altered HRV in COPD. However, the results are inconsistent across studies. β-agonist inhalation has been reported to increase sympathetic modulation of cardiovascular autonomic balance in healthy subjects or asthmatic patients [18,19]. In contrast, another study reported that HRV was not influenced by β-agonist or muscarinic antagonist [20]. There is similar controversy about the effect of COPD severity on HRV. Some studies reported that moderate or severe COPD was associated with altered autonomic function, while other studies showed that COPD severity was not related with autonomic dysfunction and HRV alteration [21,22,23,24].

Traditionally HRV is derived from an analysis of changes in R-R interval from a clinical-grade electrocardiogram (ECG) using chest wall and limb leads. However, the use of 12-lead ECG can be cumbersome and expensive. Recently, less obtrusive wearable biosensors for HRV have become popular through consumer health and wellness initiatives [25]. The HRV platform allows patients with respiratory diseases to monitor themselves at home. However, there is limited research on the feasibility of HRV measured by a wearable device in COPD patients and specifically how it correlates with various phenotypic measures of COPD.

The purpose of this paper is twofold: Firstly, to demonstrate the feasibility of ascertaining HRV via a chest-worn wearable biosensor in patients with COPD, and secondly, to use data from a clinical observational study of participants with moderate to very severe COPD, to explore associations between HRV and various phenotypic measures that relate to health and functional status.

## 2. Materials and Methods

### 2.1. Study Population

We report data from an observational study of participants with stable COPD. The participants were identified at the outpatient clinics of Ronald Reagan UCLA Medical Center and Santa Monica UCLA Medical Center. The enrollment period was from October 2016 to September 2018. Inclusion criteria were as follows: ≥40 and ≤80 years of age; smoking history > 10 pack years; ratio of forced expiratory volume in one second to forced vital capacity (FEV_1_/FVC) < 0.7; stable COPD medications for three months before enrollment, with no use of systemic corticosteroids within that time period; and no history of exacerbations within the preceding three months. The study was approved by the UCLA Institutional Review Board (IRB# 14-000748), and written informed consent was obtained from all participants.

### 2.2. Heart Rate Variability

Participants were asked to avoid all food intake, caffeine, alcohol, smoking, and heavy physical activity for 12 h before testing to control for confounding factors that could alter HRV. The HRV testing was performed between 8:00 and 11:00 a.m. with participants comfortably seated in a temperature-controlled (22 °C) room with dimmed lighting and absent distraction from noise. HRV was acquired with a commercially available, wireless, wearable multi-sensor system that included a removable puck-shaped physiological status monitor affixed to a conducive ECG fabric chest-strap (BioHarness-3^TM^, Zephyr Technologies, Annapolis, MD, USA). The puck houses the power source, transmitter, memory, and sensors that include a single channel electrocardiograph and circuitry producing R-R interval (ms). Calculations were performed at a sampling rate of 250 Hz. The lightweight Bioharness (85 g) has been shown to provide reliable measurements of heart rate compared with standard clinical-grade ECGs (SEM 2.11–5.90/min; *r* = 0.74–0.99; ICC 0.85–0.98) [26,27]. Furthermore, the Bioharness captures, logs, visualizes, and transmits the data via a Bluetooth-enabled smartphone device. After being fitted with the Bioharness on the chest at the lower sternum, participants were asked to quietly sit and breathe normally for 3 min after which the smartphone application was started and allowed to capture 7-min of R-R interval data.

Subsequently it was exported for data cleaning and analysis of heart rate variability using Kubios HRV Premium Software (Version 2.0; Biosignal Analysis and Medical Imaging Group, Department of Physics, University of Kuopio, Kuopio, Finland). HRV quantification followed the recommendations of the Task Force of the European Society of Cardiology and the North American Society of Pacing and Electrophysiology [1]. Visual inspection of the raw R-R intervals by a trained research associate was conducted to examine the presence of artifacts and an automated artifact correction algorithm from Kubios HRV was used so that artifacts and non-sinus beats could be replaced by interpolation from adjacent normal R-R intervals. The spectrum for these R-R intervals was calculated with the Welch’s periodogram method (fast Fourier transform spectrum) with a window width of 256 s and overlap of 50%. All data had less than 5% of artefacts. The cleaned signal was then used to provide normal-to-normal (N-N) intervals to compute time and frequency domain HRV parameters. For the time domain measures, we used the SDNN (standard deviation of the mean of all normal R-R intervals) which reflects all oscillatory components responsible for heart rate variability. The spectral components of low frequency (LF, 0.04–0.15 Hz) and high frequency (HF, 0.15–0.40 Hz) in normalized units (nu) were used for the frequency domain. Because the HF band represents parasympathetic activity and corresponds to the heart-rate variations related to the respiratory cycle, the ECG-derived respiration (EDR) from the Kubios HRV software excluded those outside the HF band range (0.15–0.40 Hz). The LF component is thought to be modulated by both parasympathetic and sympathetic activity. Finally, the LF/HF ratio was used to evaluate the balance between sympathetic and parasympathetic activity.

### 2.3. Other Measurements

The medical and medication history were obtained using customized questionnaires. Any history of lung disease, cardiovascular disease, or cancer was noted. COPD medications were recorded as follows: short-acting beta-agonist (SABA); long-acting beta-agonist (LABA); long-acting muscarinic antagonist (LAMA); inhaled corticosteroid (ICS); LABA plus LAMA; LABA plus ICS; LABA plus LAMA plus ICS; and phosphodiesterase-4 (PDE4) inhibitor. Beta-sympathomimetic agonists were considered to include SABA and LABA, and muscarinic antagonists were considered to include LAMA. Standing height was assessed using a mounted stadiometer (Seca, Hanover, MD, USA; accuracy ±0.01 m) and weight was assessed using a calibrated digital scale. The body mass index (BMI) was calculated from weight in kilograms divided by the square of height in meters. The spirometry maneuvers were performed using a portable electronic spirometer that conformed with technical specifications (SpiroPro^®^, Research Technology, Inc., Philadelphia, PA, USA). The 2005 American Thoracic Society/European Respiratory Society guidelines for pulmonary function testing and interpretation were used for the conduct and interpretation of spirometry [28]. The following questionnaires were administered: modified Medical Research Council (mMRC), which is a five-item instrument to assess a participant’s degree of breathlessness in relation to physical activity [29]; Veterans Specific Activity Questionnaire (VSAQ), which contains a list of daily activities, ranked from lowest metabolic equivalent (MET) value to highest MET value (1 to 13) [30]; Medical Outcomes Trust Short Form 12 (SF-12), which represents general health-related quality of life [31]; Saint George’s Respiratory Questionnaire (SGRQ), which is a well-established instrument for assessing health status in COPD and other respiratory diseases [32]; and COPD Assessment Test (CAT), which is used for assessing disease-specific quality of life for COPD patients [33]. The 6-min walk test was performed with the participant breathing room air, in accordance with the American Thoracic Society 2002 guidelines [34]. Continuous pulse oximetry was performed, and the test was stopped if the O_2_ saturation fell below 80%. The 6-min walking distance (6MWD) was reported in meters. Maximal handgrip strength for each hand was averaged from three measurements obtained using a specialized dynamometer (Jamar; Asimow Engineering Co.; Santa Monica, CA, USA). The measurements were made at rest with the hand unsupported, with the elbow at 90° flexion, underarm and with the wrist in a neutral position. The BODE index is a multidimensional grading system comprising body mass index, degree of airflow obstruction, dyspnea, and exercise capacity, and it is used to estimate the prognosis of COPD. The BODE index was calculated by an empirical model as previously described [35].

### 2.4. Statistical Analysis

Categorical variables were expressed as a number and percentage and continuous variables were expressed as a mean ± standard deviation. The Mann–Whitney (two groups) or Kruskal–Wallis (three or more groups) test was used for comparison of HRV values between groups according to the spirometric stages of severity or medications (beta-agonist and/or muscarinic antagonist). Regarding heart rate, multivariable linear regression was used to analyze the association between HRV and concurrent use of beta-agonists and muscarinic antagonists. The association between HRV and patient reported outcomes was assessed using Pearson’s correlation test for continuous variables with normal distribution or Spearman rank correlation test for continuous variables without normal distribution and for ordinal variables. For all statistical analysis, the SPSS version 25 (SPSS Inc., Chicago, IL, USA) was used, and *p* value < 0.05 was considered statistically significant.

## 3. Results

### 3.1. Baseline Characteristics

The characteristics of the participants with COPD are presented in the Table 1. Seventy-nine participants were enrolled. Their mean age was 70.6 ± 7.2 years, and the proportion of males was 60.0%. The mean FEV_1_ of participants was 62.7 ± 23.1% of predicted value, consistent with moderate COPD severity. Health and functional status questionnaires, 6MWD, and handgrip strength showed mild-to-moderate impairment. Forty-seven (58.8%) participants had cardiovascular disease. SABA, LABA/ICS, and LAMA were used in 53.8%, 33.8%, and 32.5% of participants, respectively. The mean values of heart rate (HR), SDNN, HF, and LF/HF in participants with COPD were 71.4 ± 3.3, 58.5 ± 8.1 (ms), 40.3 ± 5.2 (nu), and 2.4 ± 0.5, respectively.

### 3.2. HRV and COPD Severity

The HRV values according to COPD spirometric stage are shown in Table 2. There were no differences in HRV values with increasing COPD severity from mild to very severe. HF showed a tendency to decrease numerically as the COPD spirometric stage worsened, but the differences did not achieve statistical significance.

### 3.3. HRV and COPD Medications (Beta-Sympathomimetic Agonists and Muscarinic Antagonists)

**Figure 1:**
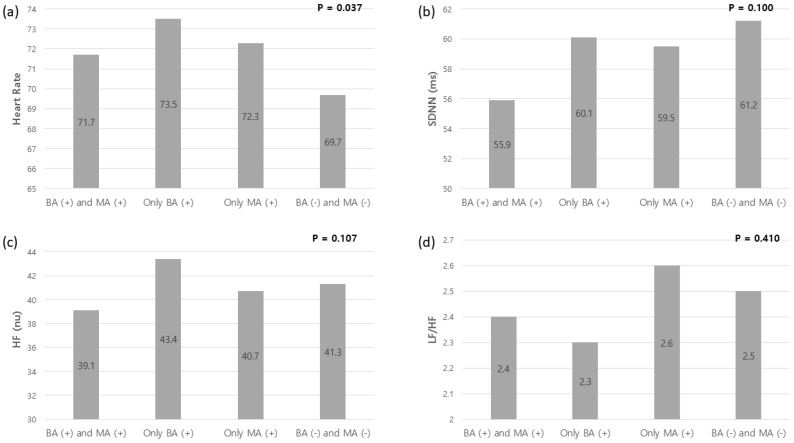
(**a**–**d**) HRV values with or without beta-agonist or muscarinic antagonist. The participants were divided into 4 groups: using concurrent beta-agonists and muscarinic antagonists (BA+ and MA+); using only beta-agonists (BA+); using only muscarinic antagonists (MA−); and using neither class of bronchodilators (BA− and MA−). HRV = heart rate variability; BA = beta-agonist; MA = muscarinic antagonist; SDNN = standard deviation of N-N interval; HF = high frequency; LF = low frequency.

The HRV values with or without beta-sympathomimetic agonists or muscarinic antagonists are shown in Figure 1. The participants were divided into four groups: using concurrent beta-agonists and muscarinic antagonists; using only beta-agonists; using only muscarinic antagonists; and using neither class of bronchodilators. Thirty-three participants used both beta-agonists and muscarinic antagonists, 20 participants did not use either, 22 participants used only beta-agonists, and four participants used only muscarinic antagonists. The SDNN tended to decrease in participants using both a beta-agonist and a muscarinic antagonist compared to those using neither class of bronchodilator drug (*p* = 0.100). The SDNN in participants using both a beta-agonist and a muscarinic antagonist was 55.9 ± 8.9 ms, whereas the SDNN in participants using neither class of bronchodilator was 61.2 ± 7.2 ms (*p* = 0.100). The SDNN in participants using only beta-agonist was not different from that in participants not using any bronchodilators (*p* = 0.190), and similar results were also found between muscarinic antagonist and no bronchodilator (*p* = 0.907). There was also no difference in SDNN between short-acting and long-acting bronchodilators (*p* = 0.538). When participants using both a beta-sympathomimetic agonist and a muscarinic antagonist were compared to those using no bronchodilators, the *p* value for SDNN was 0.021. Multivariable linear regression analysis showed that the beta coefficient for use of both a beta-sympathomimetic agonist and a muscarinic antagonist for the SDNN was −3.980 (*p* = 0.019) after adjusting for heart rate.

### 3.4. Correlation between HRV and Patient Reported Outcomes

The correlation between HRV and patient reported outcomes is shown in Table 3. Overall, the HRV had weak to moderate correlations with health or functional status in participants with COPD [36]. The SDNN was positively correlated with the VSAQ score (*r* = 0.308, *p* = 0.006) and handgrip strength (*r* = 0.285, *p* = 0.011), and negatively correlated with mMRC (*r* = −0.234, *p* = 0.039), SGRQ (*r* = −0.298, *p* = 0.008), CAT score (*r* = −0.280, *p* = 0.012), and BODE index (*r* = −0.269, *p* = 0.020), indicating poor health or functional status in subjects with lower SDNN. The HF also showed a similar correlation with health or functional status. Th HF was positively correlated with VSAQ (*r* = 0.269, *p* = 0.016), SF−12 (*r* = 0.251, *p* = 0.026), and negatively correlated with SGRQ (*r* = −0.290, *p* = 0.009) and BODE index (*r* = −0.248, *p* = 0.032), indicating poor health status in subjects with lower HF. The LF/HF was only correlated with SF-12 (*r* = 0.236, *p* = 0.036).

## 4. Discussion

Our observational study in participants demonstrates the feasibility of measuring HRV using a chest-worn wearable biosensor in patients with mild to very severe COPD. Our results confirm that HRV is reduced in this disease. We observed significant reductions in SDNN when both inhaled beta-sympathomimetic agonist and muscarinic antagonists were being taken. Decreased HRV was also associated with an increase in patient symptoms and a decrease in health and functional status.

Table 4 summarizes previous studies that analyzed HRV and patient outcomes in COPD [13,20,24,37,38,39,40,41]. A decrease in HRV has been frequently reported with alteration of autonomic nervous function in COPD. This altered HRV can be restored through exercise or rehabilitation training. However, it is controversial whether HRV is affected by the severity of COPD or inhalers such as adrenergic or anticholinergic drugs.

We have shown that HRV measured by a wearable biosensor in COPD patients, once reduced, does not appear to change with worsening COPD severity, suggesting that it is not the disease process in and of itself that reduces HRV but perhaps some other factor or factors. Previous studies have reported on the association between COPD severity and autonomic function, but the results are inconsistent. In one study of autonomic function in COPD, an abnormal blood pressure response to postural change was more frequent in moderate or severe COPD compared to mild COPD [21]. In contrast, other studies reported that parasympathetic and sympathetic dysfunction were not associated with the severity of COPD and there was no correlation of HRV with FEV_1_ [23,24].

We have shown a strong association between HRV measured by a wearable biosensor and use of inhaled bronchodilators of both the beta-sympathomimetic and muscarinic antagonist classes both when used together and alone. This is not surprising given that the intended mechanism of action of these classes of medication is to achieve bronchodilatation by targeting the autonomic nervous system. The beta-sympathomimetic agonists can cause tachycardia, palpitation, dysrhythmia, and blood pressure change, and these findings confirm that these drugs affect autonomic function [42]. Thus, in some reports, the use of beta-sympathomimetics in patients with COPD has been also associated with increased cardiovascular morbidity and mortality [43].

In patients with COPD, Bédard et al. [20] evaluated the association between HRV and beta-sympathomimetic agonists or muscarinic antagonists. These investigators reported that LF/HF ratio was significantly lower in patients with COPD compared to healthy participants, while SDNN also tended to decrease in patients with COPD. However, they found that there was no difference in LF/HF ratio for patients with COPD using or not using beta-sympathomimetic agonists or muscarinic antagonists. These results are different from those of our study. However, in the study by Bédard et al., the number of patients with COPD is small (*n* = 41), and a numerical decrease in LF/HF ratio was observed in patients using beta-sympathomimetic agonists or muscarinic antagonists (1.7 with beta-agonists versus 2.7 without beta-agonist; 1.7 with anticholinergics versus 2.7 without anticholinergics). Additionally, they did not compare patients using concurrent beta-sympathomimetic agonists and muscarinic antagonists with the other groups.

Sympathetic stimulation by short- and long-acting β-sympathomimetic agonists is known to cause tachycardia [44]. As heart rate increases, the mean R-R’ interval (heart period) decreases. Therefore, some investigators have argued that HRV metrics need to be corrected according to the mean heart period or heart rate [45]. To account for this effect, albeit small, we adjusted our measures of HRV for heart rate. Parasympathetic inhibition by short- and long-acting muscarinic antagonists can theoretically have a similar effect on heart rate. We still found that concurrent use of beta-sympathomimetic agonists and muscarinic antagonists were associated with reduced HRV after adjusting heart rate.

In our population of well-phenotyped COPD patients, we have shown that decreased HRV correlates with increased breathlessness (by mMRC), other COPD symptoms (by CAT), and worsening health status (by SGRQ). We have also shown that decreased HRV correlates with handgrip strength and decreased self-reported aerobic capacity (by VSAQ). These findings suggest that the more severe the symptoms or decreased physical activity, the more severe the impairment in the integrated autonomic function. However, our results showed that HRV was not correlated with COPD severity according to lung function based on FEV_1_. Previous studies showed that HRV, such as SDNN, LF, or HF, was correlated with daily activities or respiratory muscle strength, while the relationship between COPD severity and HRV is still controversial [1,23,24,41]. Our study also demonstrated that the BODE index is negatively correlated with SDNN and HF. The BODE index is a multi-dimensional score used to predict the survival of patients with COPD [35]. Our negative correlation with BODE suggests that HRV may help predict the risk of death in patients with COPD.

Globally, the COPD burden is expected to increase in coming decades due to continued exposure to risk factors and aging of the population [46]. The main management strategy is based on the assessment of symptom and risk of exacerbations [9]. However, exercise interventions have shown improvement in health-related quality of life, such as fatigue, which affects patient’s daily and social activities [47]. Given the impact of physical activity on the autonomic function, monitoring and quantifying the health status and physical activity of patients with COPD at home might play a crucial role for reducing their future risk. Early recognition of patients’ abnormal physiological parameters might be relevant to guide appropriate interventions and reduce healthcare utilization in patients with COPD [48], as well as optimizing quality of life [49]. For this purpose, wearable devices have the potential of continuously collecting objective and clinically meaningful data from patients at a reduced cost [50]. A previous study by Tiwari, et al. [51] reported that heart rate and physical activity data measured from wearable devices in patients with COPD were able to predict exacerbation with an area under the curve of 0.69. In contrast, Rutkowski, et al. [52] reported that wearing devices for the assessment of physical activity in patients with COPD showed no significant differences between supervised and non-supervised days. Nonetheless, they concluded that using wearable sensors in patients with COPD could be beneficial to provide feedback on quantifiable parameters, increasing the motivation to achieve health benefits. The current study aimed to explore the feasibility of using a chest-worn wearable biosensor to measure HRV in COPD patients and found a relationship between HRV, symptoms, and health and functional status. Although we demonstrated the feasibility of using wearable technology to measure HRV in COPD patients, longitudinal studies including at home measurements are needed to explore the benefits of continuous monitoring and its clinical relevance. Additional research is also needed to further explore the relationship between HRV changes and the prognosis of COPD [53,54].

Our study has limitations. There are many factors that may influence autonomic function in patients with COPD, and this study could not control for all of these factors. Examples include disease duration, exacerbation frequency, habitual physical activity, physical fitness, circadian rhythm, and other sociodemographic variables [55]. On the other hand, our study did take into account age, sex, smoking, BMI, lung function, health status, functional status, comorbidities, and COPD medications. The limitations of a large number of potentially confounding variables and correspondingly small numbers of participants are common to previous studies that analyzed HRV in patients with COPD and could be addressed by a large-scale prospective study in the future. Another limitation of our study is that serial changes in HRV and clinical features over time could not be analyzed. Although the cross-sectional design of our study does not invalidate the findings relating HRV to various clinical features of patients with COPD, the addition of serial assessment of these variables could have added useful information. In particular, future studies on how HRV affects clinical outcomes such as exacerbations, hospitalization, and death in COPD patients may be important.

## 5. Conclusions

This study confirmed that HRV is reduced in mild to very severe COPD, and HRV correlates with various health status and performance measures but is unaffected by progression of disease severity as represented by declining lung function. Current data suggest that the effects of inhaled bronchodilators on the autonomic nervous system are most likely to contribute to reduced HRV in COPD. In addition, this study reinforces the feasibility of ascertaining HRV via a chest-worn wearable biosensor in patients with COPD, as continuously monitoring physiological parameters (such as HRV and physical activity) might be crucial to assess patients’ functional status, to detect abnormal parameters early, and t guide appropriate interventions.

## Figures and Tables

**Table 1:** Baseline demographics of patients with COPD.

	Total COPD (*n* = 79)
Age (years)	70.6 ± 7.2
Male *n* (%)	48 (60.0)
Smoking (pack-years)	47.6 ± 30.5
BMI (kg/m^2^)	27.3 ± 5.1
FVC (% reference)	88.7 ± 20.6
FEV_1_ (% reference)	62.7 ± 23.1
FEV_1_/FVC	52.0 ± 14.3
Health status questionnaires	
mMRC (0–4)	1.2 ± 1.1
VSAQ (1–13)	5.5 ± 3.0
SF-12 (0–100)	64.4 ± 23.5
SGRQ (0–100)	32.7 ± 23.4
CAT (0–40)	13.6 ± 8.9
6MWD (m)	381.6 ± 133.8
Maximal handgrip strength (kg)	28.5 ± 9.9
Home oxygen	6 (7.6)
Comorbidities *n* (%)	
Cardiovascular disease	47 (58.8)
Asthma	17 (21.3)
Cancer	22 (27.5)
Medications *n* (%)	
SABA	43 (53.8)
LABA	2 (2.5)
LAMA	26 (32.5)
ICS	6 (7.5)
LABA/LAMA	4 (5.0)
LABA/ICS	27 (33.8)
LABA/LAMA/ICS	7 (8.8)
PDE4 inhibitor	2 (2.5)
HRV	
HR (/min)	71.4 ± 3.3
SDNN (ms)	58.5 ± 8.1
HFn (normalized units)	40.3 ± 5.2
LF/HF	2.4 ± 0.5

Values are presented as number (%) or mean ± standard deviation. COPD = chronic obstructive pulmonary disease; BMI = body mass index; FVC = forced vital capacity; FEV_1_ = forced expiratory volume in one second; mMRC = modified Medical Research Council; VSAQ = Veterans Specific Activity Questionnaire; SF-12 = Medical Outcomes Trust Short Form 12; SGRQ= Saint George’s Respiratory Questionnaire; CAT = COPD Assessment Test; 6MWD = 6 min walking distance; SABA = short-acting beta-agonist; LABA = long-acting beta-agonist; LAMA = long-acting muscarinic antagonist; ICS = inhaled corticosteroid; PDE4 = phosphodiesterase-4; HRV = heart rate variability; HR = heart rate; SDNN = standard deviation of N-N interval; HF = high frequency; LF = low frequency.

**Table 2:** HRV Values According to COPD Severity by Spirometric Stage.

	Grade 0 (*n* = 4)	Grade 1 (*n* = 16)	Grade 2 (*n* = 30)	Grade 3 (*n* = 23)	Grade 4 (*n* = 6)	*p* Value
HR (min)	68.3 ± 1.9	71.2 ± 3.3	71.8 ± 3.3	71.7 ± 3.8	71.4 ± 1.2	0.305
SDNN (ms)	58.6 ± 10.4	62.3 ± 5.1	56.1 ± 9.1	58.1 ± 8.3	61.6 ± 2.9	0.184
HF (normalized units)	41.1 ± 0.3	42.7 ± 4.8	39.9 ± 5.6	39.5 ± 5.3	38.8 ± 5.4	0.121
LF/HF	2.3 ± 0.1	2.5 ± 0.3	2.5 ± 0.6	2.2 ± 0.5	2.3 ± 0.4	0.205

Values are presented as mean ± standard deviation. HRV = heart rate variability; COPD = chronic obstructive pulmonary disease; HR = heart rate; SDNN = standard deviation of N-N interval; HF = high frequency; LF = low frequency.

**Table 3:** Correlation between HRV and patient reported outcomes.

	HR	SDNN	HF	LF/HF
Smoking (pack-years)	0.020	0.031	−0.080	−0.182
BMI (kg/m^2^)	−0.144	0.103	0.140	−0.132
% FVC (% reference)	−0.060	0.049	0.045	0.205
% FEV_1_ (% reference)	−0.129	0.059	0.108	0.151
Health status questionnaires				
mMRC (0–4)	0.169	−0.234 *	−0.184	−0.081
VSAQ (1–13)	−0.241 *	0.308 **	0.269 *	0.101
SF-12 (0–100)	−0.229 *	0.194	0.251 *	0.236 *
SGRQ (0–100)	0.155	−0.298 **	−0.290 **	−0.027
CAT (0–40)	0.151	−0.280 *	−0.221	0.065
6MWD (m)	−0.272 *	0.207	0.160	0.010
Handgrip strength (kg)	−0.294 **	0.285*	0.184	−0.029
BODE index	0.140	−0.269 *	−0.248 *	−0.081

Values are Pearsons or Spearman’s rank correlation coefficients. HRV = heart rate variability; HR = heart rate; SDNN = standard deviation of N-N interval; HF = high frequency; LF = low frequency; BMI = body mass index; FVC = forced vital capacity; FEV_1_ = forced expiratory volume in one second; mMRC = modified Medical Research Council; VSAQ = Veterans Specific Activity Questionnaire; SF-12 = Medical Outcomes Trust Short Form 12; SGRQ = Saint George’s Respiratory Questionnaire; CAT = Chronic Obstructive Pulmonary Disease Assessment Test; 6MWD = 6 min walking distance; BODE index (B = body mass index; O = degree of airflow obstruction; D = dyspnea; E = exercise capacity). * *p* < 0.05; ** *p* < 0.01.

**Table 4:** Previous studies about HRV and patient outcomes in COPD.

Selected Studies	Age (Years)	FEV_1_ (%)	SDNN (ms)	HF (nu)	HF/LF	Principal Outcomes
Bédard, et al. (*n* = 41)	67	45	NA	NA	1.9	HRV correlated with disease severity and did not seem to be influenced by anticholinergic or adrenergic medications.
Camillo, et al. (*n* = 31)	66	46	33	55	NA	HRV was not related to disease severity but mainly to the level of physical activity in daily life.
Bartels, et al. (*n* = 53)	61	35	NA	NA	3.1	The balance of sympathetic to parasympathetic cardiac modulation decreased in patients with COPD during maximal volitional exercise.
Camillo, et al. (*n* = 20)	67	40	29	56	0.9	High-intensity exercise training improved HRV at rest and during orthostatic stimulus in patients with COPD.
Ricci-Vitor, et al. (*n* = 13)	67	48.3	17	NA	NA	The exclusive resistance training improved sympathetic and parasympathetic components of autonomic nervous system representing by SDNN, LF, and HF.
Zupanic, et al. (*n* = 31)	61	NA	24	45	1.8	A 4-week rehabilitation improved HRV (SDNN).
Leite et al. (*n* = 37)	63	46	20	37	1.7	HRV indexes at rest was correlated with aerobic physiological variables obtained at a maximal exercise test.
Goulart, et al. (*n* = 10)	61	32	NA	54	0.9	COPD patients with impaired respiratory muscle strength showed marked sympathetic modulation and a reduced parasympathetic response; reduced HRV complexity was observed during a respiratory sinus arrhythmia maneuver.

COPD = chronic obstructive pulmonary disease; FEV_1_ = forced expiratory volume in one second; SDNN = standard deviation of N-N interval; HF = high frequency; LF = low frequency; HRV = heart rate variability.

## Data Availability

The data that support the findings of this study are available from the corresponding author, upon reasonable request.
